# Real-world treatment patterns and outcomes of patients with advanced melanoma treated with nivolumab plus relatlimab

**DOI:** 10.1093/oncolo/oyae248

**Published:** 2024-09-18

**Authors:** Sach Thakker, Micah Belzberg, Sekwon Jang, Jafar Al-Mondhiry

**Affiliations:** Georgetown University School of Medicine, Washington, DC, United States; Department of Dermatology, The Johns Hopkins University School of Medicine, Baltimore, MD, United States; Inova Schar Cancer Institute, Fairfax, VA, United States; Inova Schar Cancer Institute, Fairfax, VA, United States

**Keywords:** cutaneous oncology, melanoma, nivolumab, relatlimab, immunotherapy, immune checkpoint inhibitor, PD-1 inhibitor, LAG-3 inhibitor

## Abstract

The use of programmed death-1 (PD-1) inhibitors has been a pivotal advancement in treating advanced melanoma, yet their efficacy is limited. The approval of relatlimab (RELA), a lymphocyte activation gene 3 protein (LAG-3) antibody, in combination with nivolumab (NIVO), a PD-1 inhibitor, marked a significant stride toward enhancing treatment efficacy for metastatic and unresectable stage 3 and 4 melanoma. This combination has been shown to synergistically improve antitumor activity and effector T-cell activity in the tumor microenvironment, despite limited data on real-world outcomes. Our retrospective review at a tertiary cancer center of patients with stage 3 and 4 melanoma treated with NIVO-RELA revealed an overall response rate (ORR) of 39%, with notable improvements in median PFS and ORR, especially in first-line treated patients. Our study highlights the superior efficacy of NIVO-RELA over previous reports, demonstrating its significant potential in the treatment landscape of advanced melanoma.

## Teaser

This brief communication highlights the superior efficacy of NIVO-RELA over previous reports, demonstrating its significant potential in the treatment landscape of advanced melanoma.

Programmed death-1 (PD-1) inhibitors have significantly improved outcomes for patients with advanced melanoma. However, overall response rates and progression-free survival with first-line PD-1 inhibitor therapy remain limited to 32.6% and 4.6 months, respectively.^1^ Relatlimab (RELA), a lymphocyte activation gene 3 protein (LAG-3) antibody, was approved in 2022 by the US Food and Drug Administration for the treatment of metastatic and unresectable stage 3 and 4 melanoma in combination with nivolumab (NIVO), an existing PD-1 inhibitor.^2,3^ The NIVO-RELA combination results in synergistic antitumor activity with increased effector T-cell activity in the tumor microenvironment.^4^ Although clinical trials have demonstrated NIVO-RELA provides benefit for patients with advanced melanoma, little is known about real-world treatment patterns and outcomes.^1-5^

We performed a retrospective review of all patients with stage 3 and 4 melanoma treated with NIVO-RELA as first- (1L) or second-line or beyond (2L+) therapy at Inova Schar Cancer Institute between September 2021 and September 2023. Patients were excluded if they were followed for less than 1 month following NIVO-RELA initiation; however, patients who expired within 1 month of NIVO-RELA initiation were included. Given the retrospective nature of this study, accurate toxicity reporting was not available. Primary endpoints were overall response rate (ORR), complete response (CR), partial response (PR), stable disease, and progression of disease (PD) which were evaluated using the RECIST v1.1 criteria. Subgroup outcome analyses were performed. ORR, OS, and PFS were computed with log-rank tests. The Kaplan-Meier method was used to generate OS and PFS median values and 95% CIs. Hazard ratios (HRs) were calculated with Cox proportional hazards models.

**Figure 1.**
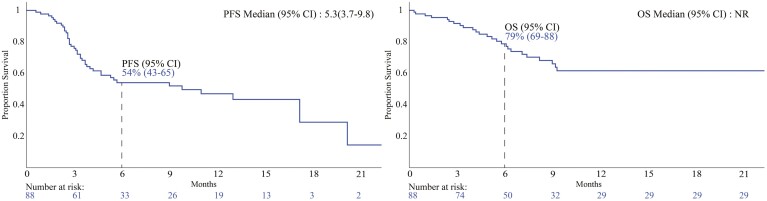
Overall cohort progression-free survival (PFS) and overall survival (OS) analyses shown with Kaplan-Meier survival curves. Time in months.

Eighty-eight patients were followed for an average of 6.5 months following NIVO-RELA initiation (Supplementary Table S1). The entire cohort had an ORR of 39%, with 16% PR and 23% CR. Median PFS was 5.3 months (95% CI 3.7-9.8) (Figure 1). For 1L treated patients, ORR was 58%, with 21% PR and 37% CR. Median PFS was 11 months (95% CI 3.1-11). For 2L+ treated patients, ORR was 33%, with 14% PR and 19% CR. Median PFS was 9.8 months (95% CI 4.7-17.2) (Supplementary Figures S1 and S2). Subgroup analyses observed significantly different ORR between patients previously treated with versus without anti-CTLA4 therapy (29% vs 44%; *P* = .017), patients treated with NIVO-RELA more than 6 months from their last other therapy versus within 6 months from their last other therapy (50% vs 27%; *P* = .004), and among patients with stage 3 versus 4 disease (67% vs 30%; *P* = .040) (Table 1). PFS did not significantly differ in subgroup analyses (Supplementary Figures S1-S3). However, stage 4 patients treated 1L with NIVO-RELA displayed significantly greater OS compared to stage 4 patients treated with NIVO-RELA as 2L or more line therapy (HR 2.75; median OS not reached; *P* = .045).

**Table 1.** Subgroup analyses.

Group 1	Group 2	Group 1				Group 2				OR
		*N*	PR	CR	OR	*N*	PR	CR	OR	*P*-value
1L therapy	2L+	19	21%	37%	58%	69	14%	19%	33%	.058
BRAF mutation	No mutation	30	27%	13%	40%	58	10%	28%	38%	.582
Prior anti-PD-1	No prior	67	15%	19%	34%	21	19%	33%	52%	.083
<6 months since prior PD-1	>6 months	32	6%	25%	31%	35	23%	14%	37%	.325
Prior anti-CTLA4	No prior	34	18%	12%	29%	54	15%	30%	44%	**.017**
Prior anti-BRAF/MEK	No prior	19	21%	16%	37%	69	14%	25%	39%	.185
<6 months since prior treatment	>6 months	49	8%	18%	27%	20	30%	20%	50%	**.004**
Stage 4	Stage 3	63	14%	16%	30%	21	24%	43%	67%	**.040**
Stage 3	Not stage 3	21	24%	43%	67%	67	13%	16%	30%	**.031**
Stage 4	Not stage 4	63	14%	16%	30%	25	20%	40%	60%	.084
Stage 4 1L	Stage 4 2L+	10	30%	30%	60%	53	11%	13%	25%	.070
Stage 4 cutaneous	Stage 4 acral	47	17%	17%	34%	8	0%	25%	25%	.519
Stage 4 cutaneous	Stage 4 mucosal	47	17%	17%	34%	5	0%	0%	0%	.159
Stage 4 acral	Stage 4 mucosal	8	0%	25%	25%	5	0%	0%	0%	.228

OR *P*-value calculated with log-rank test. Statistically significant *P*-values bolded.

Abbreviations: OR, overall response; PR, partial response; CR, complete response; m, months.

RELATIVITY-047 showed an ORR of 43% and a median PFS of 10.4 months as a 1L treatment while RELATIVITY 020 showed an ORR of 9%-12% and a median PFS of 2.1-3.2 months as a 2L+ treatment.^4,5^ These results support the use of NIVO-RELA in patients with advanced melanoma and emphasize the impressive efficacy of the drug combination in the 1L and 2L+ settings. PFS and OS for 1L and 2L+ therapy with NIVO-RELA in this study aligned with or exceeded results from previous trials. This study was limited by the retrospective nature of the analysis, incomplete data and/or potential misclassification, limited follow-up time, and the relatively small sample sizes. Further analyses with increased sample sizes and longer follow-ups are warranted.

## Supplementary Material

Supplementary material is available at *The Oncologist* online.

oyae248_suppl_Supplementary_Material

oyae248_suppl_Supplementary_Material

## Data Availability

The data underlying this article will be shared on reasonable request to the corresponding author.
