# Supplementary material for: Real-world treatment patterns and outcomes of patients with advanced melanoma treated with nivolumab plus relatlimab
Source: Oncologist. 2024 Sep 18;29(12):e1783–5. doi: 10.1093/oncolo/oyae248 (PMC11630778; doi:10.1093/oncolo/oyae248)
Supplement: oyae248_suppl_Supplementary_Material [file oyae248_suppl_supplementary_material.pdf]

Supplemental Table 1: Study demographic data.

	N(%) or Med
Patients, n	88
Female, n(%)	40(45.5%)
Male, n(%)	48(45.5%)
Age at index, median (range)	66(22-88)
Follow up, median months	6.5
**Race**	
White, n(%)	72(81.8%)
AA, n(%)	4(4.5%)
Other, n(%)	12(13.6%)
**Location**	
Cutaneous	64(72.7%)
Acral	10(11.4%)
Mucosal	10(11.4%)
Unkown	4(4.5%)
**Stage**	
Stage III, n(%)	21(23.9%)
Stage IV, n(%)	63(71.6%)
Brain metastasis, n(%)	21(23.9%)
BRAF mutation, n(%)	30(34.1%)
**Treatment**	
1st Line Tx, n(%)	19(21.6%)
Prior PD1, n(%)	67(76.1%)
<6m since prior PD1, n(%)	32(36.4%)
Prior CTLA4, n(%)	34(38.6%)
Prior BRAF/MEK, n(%)	19(21.6%)
Prior Treatments, n(%)	
1, n(%)	27(30.7%)
2, n(%)	25(28.4%)
3, n(%)	7(8%)
4, n(%)	4(4.5%)
>4, n(%)	6(6.8%)
<6m since prior Tx, n(%)	49(55.7%)
**Outcome**	
Overall response, n(%)	34(38.6%)
Partial response, n(%)	14(15.9%)
Complete response, n(%)	20(22.7%)
Progression, n(%)	42(47.7%)
Deceased, n(%)	25(28.4%)
