# Supplementary material for: Real-world treatment patterns and outcomes of patients with advanced melanoma treated with nivolumab plus relatlimab
Source: Oncologist. 2024 Sep 18;29(12):e1783–5. doi: 10.1093/oncolo/oyae248 (PMC11630778; doi:10.1093/oncolo/oyae248)

**Supplemental Figure Captions**

**Supplemental Figure 1:** Kaplan-Meier survival curves for subgroup analyses comparing 1st v 2nd line therapy, presence of BRAF mutation, prior Anti-PD1 treatment, <6 months from prior PD1 treatment, prior anti CTLA4 treatment, and BRAF/MEK therapy. Time in months. PFS, progression free survival; OS, overall survival; 95% CI, 95% confidence interval; HR, hazards ratio; NR, not reached.


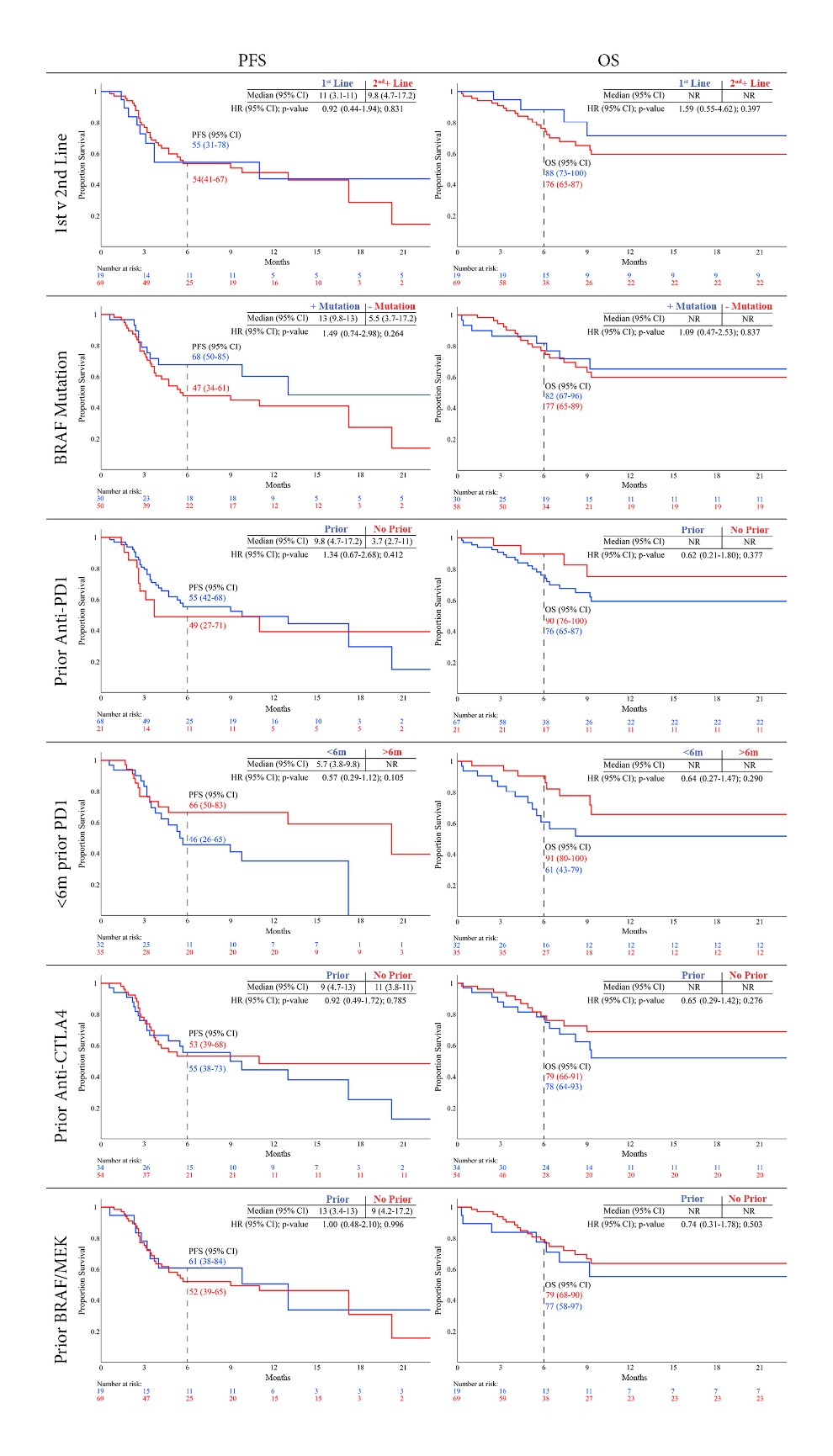


**Supplemental Figure 2:** Kaplan-Meier survival curves for subgroup analyses comparing prior <6m from prior therapy, Stage IV melanoma or Stage III melanoma, Stage III melanoma, Stage IV melanoma, and Stage IV melanoma treated with NIVO-RELA as 1st line v 2nd+ line. Time in months. PFS, progression free survival; OS, overall survival; 95% CI, 95% confidence interval; HR, hazards ratio; NR, not reached.


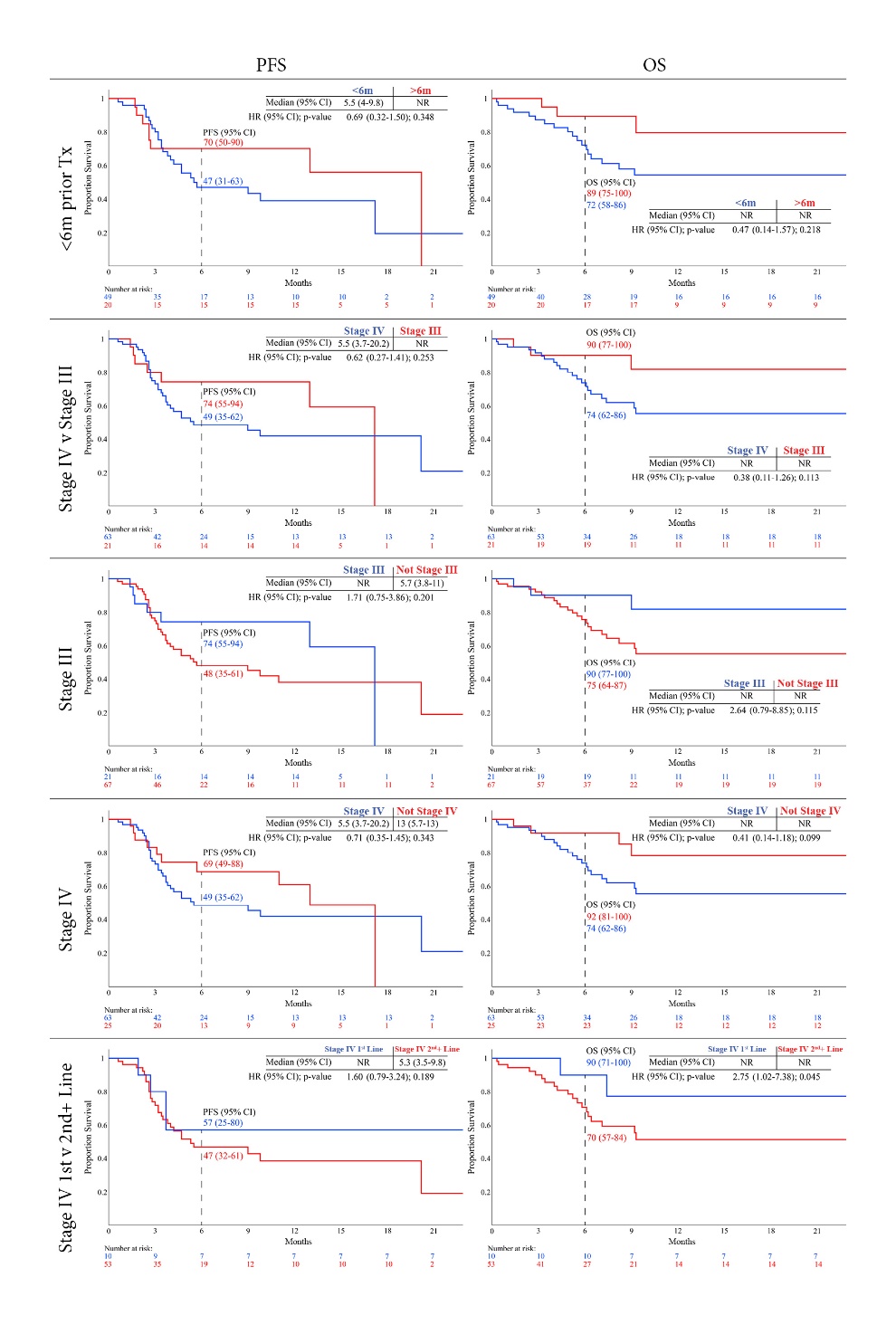


**Supplemental Figure 3:** Kaplan-Meier survival curves for subgroup analyses comparing prior Stage IV cutaneous melanoa v acral melanoma, Stage IV cutaneous melanoma, mucosal melanoma, and Stage IV acral melanoma v mucosal melanoma. Time in months. PFS, progression free survival; OS, overall survival; 95% CI, 95% confidence interval; HR, hazards ratio; NR, not reached.


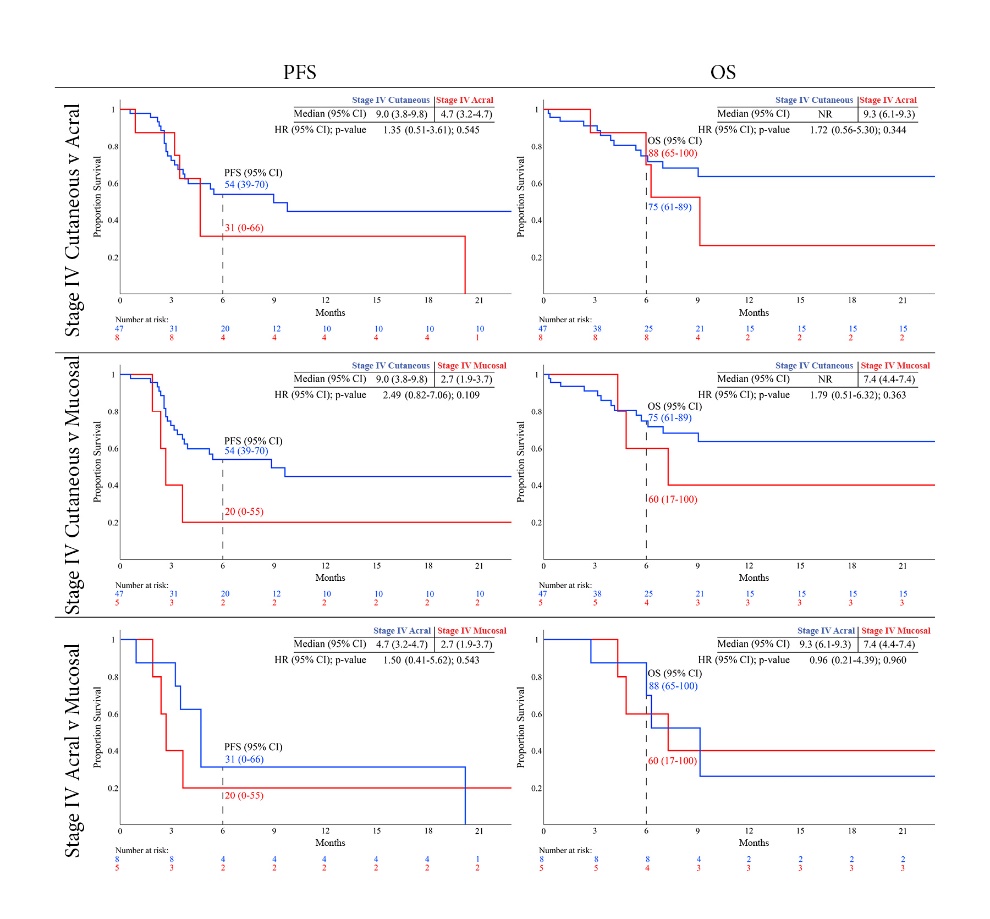

Supplement: oyae248_suppl_Supplementary_Material [file oyae248_suppl_supplementary_material.docx]
